# Therapeutic use of cannabis and cannabinoids: an evidence mapping and appraisal of systematic reviews

**DOI:** 10.1186/s12906-019-2803-2

**Published:** 2020-01-15

**Authors:** Nadia Montero-Oleas, Ingrid Arevalo-Rodriguez, Solange Nuñez-González, Andrés Viteri-García, Daniel Simancas-Racines

**Affiliations:** 1 Centro de investigación en Salud Pública y Epidemiología Clínica (CISPEC). Facultad de Ciencias de la Salud “Eugenio Espejo”, Universidad UTE, Quito, Ecuador; 2 Clinical Biostatistics Unit, Hospital Ramon y Cajal (IRYCIS), CIBER of Epidemiology and Public Health, Madrid, Spain

**Keywords:** Cannabis, Cannabinoids, Medical marijuana, Evidence mapping, Evidence synthesis

## Abstract

**Background:**

Although cannabis and cannabinoids are widely used with therapeutic purposes, their claimed efficacy is highly controversial. For this reason, medical cannabis use is a broad field of research that is rapidly expanding. Our objectives are to identify, characterize, appraise, and organize the current available evidence surrounding therapeutic use of cannabis and cannabinoids, using evidence maps.

**Methods:**

We searched PubMed, EMBASE, The Cochrane Library and CINAHL, to identify systematic reviews (SRs) published from their inception up to December 2017. Two authors assessed eligibility and extracted data independently. We assessed methodological quality of the included SRs using the AMSTAR tool. To illustrate the extent of use of medical cannabis, we organized the results according to identified PICO questions using bubble plots corresponding to different clinical scenarios.

**Results:**

A total of 44 SRs published between 2001 and 2017 were included in this evidence mapping with data from 158 individual studies. We extracted 96 PICO questions in the following medical conditions: multiple sclerosis, movement disorders (e.g. Tourette Syndrome, Parkinson Disease), psychiatry conditions, Alzheimer disease, epilepsy, acute and chronic pain, cancer, neuropathic pain, symptoms related to cancer (e.g. emesis and anorexia related with chemotherapy), rheumatic disorders, HIV-related symptoms, glaucoma, and COPD. The evidence about these conditions is heterogeneous regarding the conclusions and the quality of the individual primary studies. The quality of the SRs was moderate to high according to AMSTAR scores.

**Conclusions:**

Evidence on medical uses of cannabis is broad. However, due to methodological limitations, conclusions were weak in most of the assessed comparisons. Evidence mapping methodology is useful to perform an overview of available research, since it is possible to systematically describe the extent and distribution of evidence, and to organize scattered data.

## Background

Medical cannabis refers to the use of cannabis or cannabinoids for the treatment of a medical condition or to alleviate its associated symptoms [1, 2]. The spectrum of substances categorized as medical cannabis include: 1) Phytocannabinoids, which are found in cannabis herb and resins, e.g. Tetrahydrocannabinol (THC) and Cannabidiol (CBD); 2) Purified cannabinoids which originate from cannabis extracts (e.g. Nabiximols and purified cannabidiol); and 3) Synthetic cannabinoids (e.g. Dronabinol and Nabilone) [2, 3].

*Cannabis sativa* produces more than 100 phytocannabinoids and the biosynthesis of these substances depends on genomic background and specific environmental conditions [4]. Additionality, in humans, the use of *C. sativa* has shown a myriad of heterogeneous central and peripheral effects due to endocannabinoid system, whose receptors are scattered throughout the body. The existence of many molecules, which possibly modulate endocannabinoid system, complicates the scenario [5]. Currently, these are the reasons why, research on *C. sativa* is complex and difficult.

The history of the use of cannabis for medical purposes is long, as these plants have been used for therapeutic purposes for more than 4000 years [6]. However, cannabis has a high-risk profile and its medical use is highly controversial, even for therapeutic reasons. Despite the adverse effects of cannabis use such as risk of developing cannabis dependence, exacerbation of cardiovascular disease, precipitation of psychotic disorders [7], and criticism to the evidence supporting its use for medical conditions, several governments have authorized the medical use of marijuana in countries such as Canada, the Czech Republic, Germany, Italy, the Netherlands, and 23 US states [8–10].

To approve the medical use of cannabis, well-designed and statistically powered clinical trials are necessary to investigate patient response [11]. Research on therapeutic uses of cannabis have restrictions due to limitations in gaining access to the quantity, quality, and type of cannabis product necessary to address specific research questions on health effects. There are notable research challenges, such as the vast spectrum of chemical substances considered as medical cannabis, the lack of dose standardization, and the lack of consensus about medical conditions for which cannabis have been approved. Evidence about the benefits and harms related to cannabis use is rapidly changing, making it difficult to identify and summarize findings in order to make informed decisions and establish research needs.

Evidence mapping is a useful methodology to overview available research about broad knowledge areas. This methodology is useful to systematically describe the extent and distribution of evidence and to identify gaps for further research. This approach identifies if there is enough evidence to support policy maker’s decisions and to recognize research-dense areas where systematic reviews can be conducted, as well as research questions which should be prioritized in those fields.

The aim of this evidence mapping is to identify, characterize, appraise, and organize the currently available evidence about the therapeutic use of cannabis and cannabinoids through systematic reviews. Our approach aims to identify the clinical questions about efficacy of medical cannabis assessed in the scientific literature, as well as to give an overview about their potential benefits and harms.

## Methods

We followed the approach of the Global Evidence Mapping Initiative [12] with additional components introduced by Ballesteros et al. [13–15]. We established these criteria a priori in a protocol (available on request). This evidence map involved three stages:

### Systematic search strategy and selection of relevant studies

We used systematic reviews (SRs) as a comprehensive source of appraised evidence. We defined medical cannabis as the use of cannabis or cannabinoids to treat a medical condition or to alleviate its symptoms. Thus, we based this evidence mapping in SRs assessing medical cannabis efficacy, effectiveness or safety. We decide to include cannabis and cannabinoids as our objective is to identify all the available evidence related to medical cannabis, however cannabis and their isolated compounds could have different pharmacological properties and efficacy profiles. We considered SRs that conducted a search in at least two databases, and that appraise the quality or risk of bias of the included studies.

We excluded SRs focused on cost-effectiveness only. Additionally, we excluded SRs assessing Rimonabant (i.e. a synthetic cannabinoid studied for weight control) since it acts as a functional antagonist of cannabinoids receptor [16].

We collected key search terms from previous reviews and SRs on medical cannabis by using natural and MeSH terms. We searched in PubMed, EMBASE, The Cochrane Library and CINAHL, from their inception up to December 2017. There were no language restrictions.

We reviewed references in relevant articles to identify potential additional reviews. Search strategies are reported in Additional file 1.

After duplicates were eliminated, two reviewers independently screened titles and abstracts (NMO, SNG) of the retrieved references and determined their relevance according to the eligibility criteria. On a second stage, full-texts of potentially relevant reviews were obtained for a final decision. Disagreements were resolved through discussion; if necessary, a third reviewer was consulted.

### Data extraction of the included SRs

For included SRs, we collected data about their general characteristics, as well as data about the gathered information from individual studies. For data extraction, each reviewer went through a pilot test to standardize the process. We designed an extraction form to collect data at three levels:

#### Characteristics of included SRs and methodological quality

We collected data about author(s), year of publication, search date, searched databases, objective, design, number of included studies and patients, and methods used for the assessment of risk of bias.

Two reviewers independently assessed the methodological quality of the included SRs by using the AMSTAR tool [17]. Disagreements were discussed until consensus was reached. We calculated a global AMSTAR score assigned one point for each item rated as “yes” and items rated as “no”; “cannot answer”, or “not applicable” obtained zero points, resulting in an overall score ranging from 0 to 11. Based on the reported score we classified each SR into three categories: low (0 to 3 points), moderate (4 to 7 points), and high quality (8 to 11 points) [17].

#### Clinical questions assessed in the SRs

We collected information related to research questions in PICO format (e.g. Population, Intervention, Comparator and Outcomes). For descriptive purposes, we categorized conclusions reported by authors for each PICO question, into six categories: “unclear”, “no effect”, “probably harmful”, “harmful”, “probably beneficial” and “beneficial”, as the categorization performed in previous evidence mapping. See Table 1, for further details of the category definition. Two reviewers independently categorized the conclusions. Discrepancies were discussed until consensus was reached. In all cases, judgement represented a formal assessment about the evidence, benefits and harms of each intervention.

**Table 1:** Classification of the conclusions according to results reported by authors

Classification	Definition
Unclear	Direction of results differed within reviews due to conflicting results or limitations of individual studies.
No effect	The conclusions provided evidence of no difference between intervention and comparator.
Probably harmful	The conclusions did not claim for firm harmful effect despite the reported negative treatment effect.
Harmful	The conclusions were reported as clearly indicative of a harmful effect.
Probably beneficial	The conclusions did not claim for firm benefits despite the reported positive treatment effect.
Beneficial	The conclusions reported a clear beneficial effect without major concerns regarding the supporting evidence.

#### Characteristics of individual studies included in SRs

We collected the following information about the individual studies included in each SR: abstract, number of included patients, country, funding, follow-up, type of study, condition, intervention, comparison, and methodological quality according to the authors of the SRs.

### Synthesize the results into a user-friendly format

We presented our findings on tables and figures to describe the characteristics of the included SRs. Additionality, we classified the information according to PICO questions. Thus, for each PICO we obtained the number of SRs, individual studies, and patients.

We mapped the extent of the evidence using bubble plots. Each bubble represents one SR. The chart displays information using three dimensions: (i) Authors conclusions (“unclear”, “no effect”, “probably harmful”, “harmful”, “probably beneficial” and “beneficial”) in the x-axis; (ii) Score from AMSTAR assessment in the y-axis, and (iii) The number of participants included in the SR assessing the PICO question represented in the bubble size. Systematic reviews may have been represented more than once in the plot as one SR could have answered different PICO questions.

## Results

**Fig. 1:**
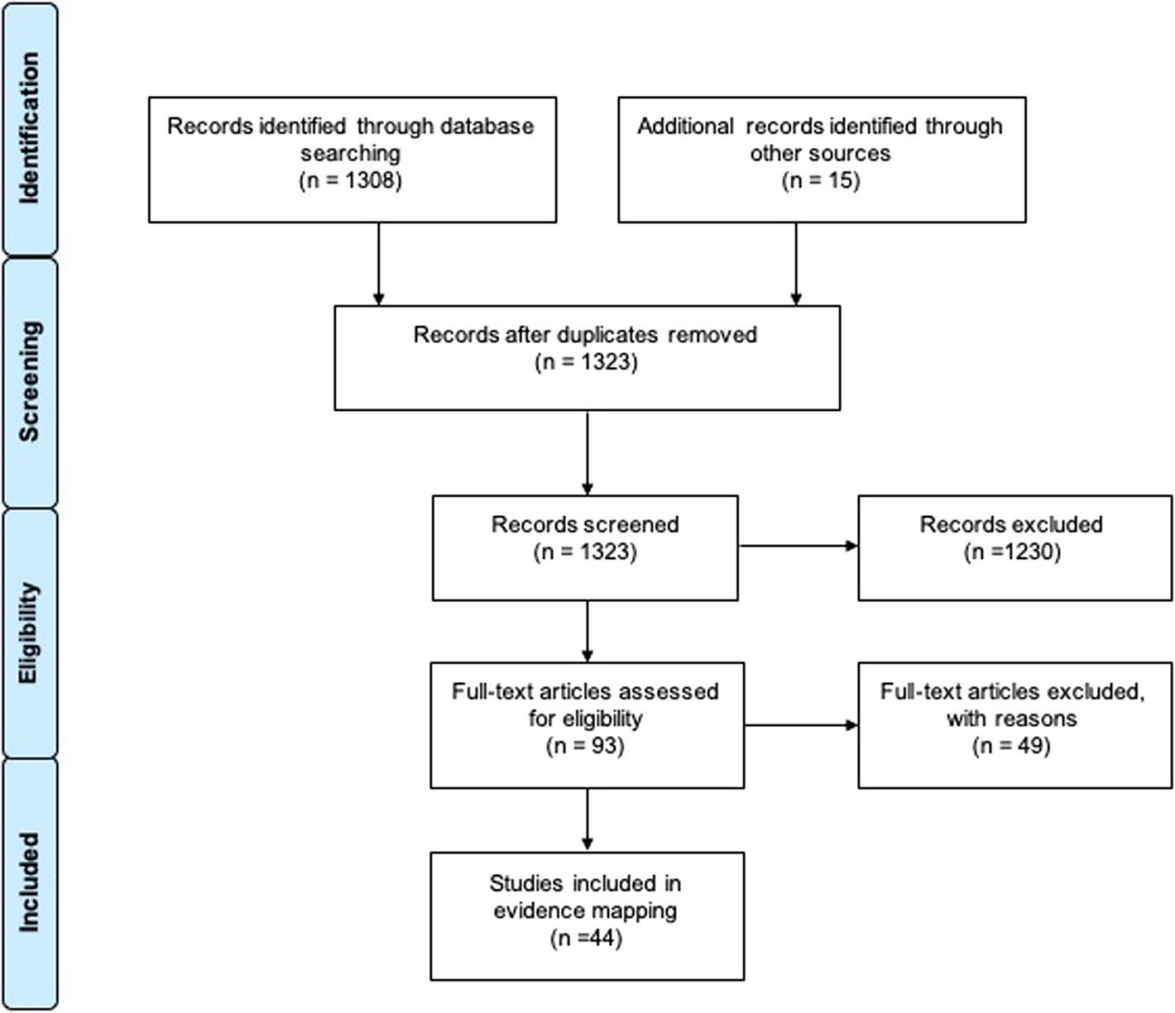
Flow chart outlining the study selection process

We obtained a total of 1323 records after duplicates were removed. Following titles and abstracts screening, 93 articles were obtained in full-text for a final decision. We included a total of 44 SRs in the final selection (Fig. 1). A list of excluded reviews with exclusion rationale is available in Additional file 2.

### Characteristics and quality of systematic reviews

Included SRs were published between 2001 and 2017 comprising studies conducted between 1975 and 2016. The last search was conducted in November 2016 [18]. All but one SR assessed the effectiveness of cannabis or cannabinoids, while the remaining SR evaluated the cannabinoids adverse events only [19]. Seventeen out of 44 included SRs performed a meta-analysis of data. See Table 2 for additional characteristics of SRs.

**Table 2:** Characteristics of systematic reviews included in the Evidence Mapping

Systematic review	Search date	Population	Included studies	Studies design				Number of patients	Meta-analysis	Tool for risk of bias assessment
				RCT	NRCT	UCT	Obs			
ABO 2017	November 2016	Neurogenic urinary tract dysfunction in multiple sclerosis (MS).	3	2	0	1	0	426	Yes	Cochrane Risk of Bias
ANDREAE 2015	April 2014	Chronic neuropathic pain	5	5	0	0	0	178	Yes	Cochrane Risk of Bias
BALDINGER 2012	February 2011	Amyotrophic lateral sclerosis/ motor neuron disease	1	1	0	0	0	22	No	Cochrane Risk of Bias
BEGA 2014	April 2014	Parkinson Disease	2	2	0	0	0	26	No	American Academy of Neurology classification
BOYCHUCK 2015	April 2013	Chronic nonmalignant neuropathic pain	13	13	0	0	0	771	No	JADAD 5
CAMPELL 2001	October 1999	Pain.	8	6	0	0	2	222	No	JADAD 5
CURTIS 2009	October 2008	Gilles de la Tourette	2	2	0	0	0	28	No	CONSORT
DE SOUZA 2013	December 2012	Fibromyalgia	2	2	0	0	0	69	No	Jadad5, Cochrane
DESHPANDE 2015	April 2014	Chronic noncancer pain	6	6	0	0	0	226	No	JADAD 5
FINNERUP 2015	April 2013	Neuropathic pain	9	9	0	0	0	1310	Yes	JADAD 5
FITZCHARLES 2016a	April 2015	Fibromyalgia, back pain, osteoarthritis, rheumatoid arthritis	4	4	0	0	0	160	No	Cochrane Risk of Bias
FITZCHARLES 2016b	January 2015	Rheumatic diseases	4	4	0	0	0	201	No	Cochrane Risk of Bias
GLOSS 2014	September 2013	Epilepsy	4	4	0	0	0	48	No	Cochrane Risk of Bias
ISKEDIJAN 2006	June 2006	Neuropathic pain.	7	7	0	0	0	298	Yes	JADAD 5
JAWAHAR 2013	December 2012	Multiple sclerosis patients.	4	4	0	0	0	565	Yes	Cochrane Risk of Bias
KHAISER 2016	May /2016	Pain	11	9	0	1	1	420	No	Own criteria
KOPPEL 2014	November 2013	Several neurologic conditions	28	26	1	1	0	3567	No	American Academy of Neurology classification
KRISHNAN 2009	April 2008	Dementia	1	1	0	0	0	15	No	Cochrane Risk of Bias
LAKHAN 2009	April 2009	Multiple Sclerosis-related spasticity	6	6	0	0	0	481	No	JADAD 5
LANGHORST 2015	March 2014	Inflamatory Bowel Disease	1	1	0	0	0	22	No	Cochrane Risk of Bias
LUTGE 2013	July 2012	Patients infected with human immunodeficiency virus (HIV)	7	7	0	0	0	330	No	Cochrane Risk of Bias
LYNCH 2011	September–October 2010	Chronic pain	18	18	0	0	0	766	No	JADAD 7
LYNCH 2015	October 2014	Chronic pain	11	0	0	0	0	1185	No	JADAD 7
MACHADO 2008	December 2006	Cancer patients receiving chemotherapy	30	30	0	0	0	1719	Yes	JADAD
MARTIN 2009	February 2008	Chronic pain.	18	18	0	0	0	809	Yes	JADAD 5
MEHTA 2015	September 2015	Pain post spinal cord injury	2	2	0	0	0	29	No	PEDRO
MEYER 2010	2008	Acute phase of acquired brain injury	2	2	0	0	0	928	No	PEDRO
MÜCKE 2016	April 2015	Palliative medicine	9	9	0	0	0	1561	Yes	Cochrane Risk of Bias
OMS 2016	September 2016	Multiple sclerosis, chronic pain, HIV/AIDS, Dementia, Tourette syndrome and adults in chemotherapy.	43	43	0	0	0	4586	Yes	Cochrane Risk of Bias
OTERO-ROMERO 2016	August 2013	Multiple Sclerosis-related spasticity	8	8	0	0	0		No	EFNS scientific task forces
PETZKE 2016	November 2015	Neuropathic pain syndromes	15	15	0	0	0	1619	Yes	Cochrane Risk of Bias
PILLIPS 2010	February 2010	Neuropathic pain in HIV patients	2	2	0	0	0	89	Yes	JADAD 7
PILLIPS 2016	December 2014	Children and young people receiving chemotherapy	4	4	0	0	0	78	Yes	Cochrane Risk of Bias
RICHARDS 2012	December 2010	Pain in patients with rheumatoid arthritis	1	1	0	0	0	58	No	COChrane Risk of Bias
SHAKESPEARE 2003	June 2003	Multiple Sclerosis-related spasticity	2	2	0	0	0	40	No	Cochrane Risk of Bias
SMITH 2015	January 2015	Chemotherapy-induced nausea in cancer patients	23	23	0	0	0	1326	Yes	Cochrane Risk of Bias
SNEDECOR 2014	June 2011	Painful diabetic peripheral neuropathy	1	1	0	0	0	30	Yes	JADAD 5
STEVENS 2017	August 2016	Acute pain.	7	7	0	0	0	611	No	Cochrane Risk of Bias
TEASELL 2010	June 2009	Spinal Cord Injury	2	1	0	0	1	22	No	PEDRO, Downs and Black checklist
TRAMER 2001	August 2008	Sickness induced by chemotherapy	30	30	0	0	0	1366	Yes	JADAD 5
VAN DEN ELSEN 2014	October 2013	Older subjects.	5	5	0	0	0	267	No	Cochrane Risk of Bias
VOLZ 2016	March 2015	Inflammatory diseases, irritable bowel syndrome and chronic pancreatitis	1	1	0	0	0	21	No	Cochrane Risk of Bias
WANG 2008	October 2007	Safety of medical cannabis use.	31	23	0	0	8	3107	Yes	JADAD, Downs and Back checklist
WHITING 2015	April 2015	Chronic pain, spasticity, adults in chemotherapy, weight gain in HIV, sleep disorders, Tourette syndrome	79	79	0	0	0	6462	Yes	Cochrane Risk of Bias

**Fig. 2:**
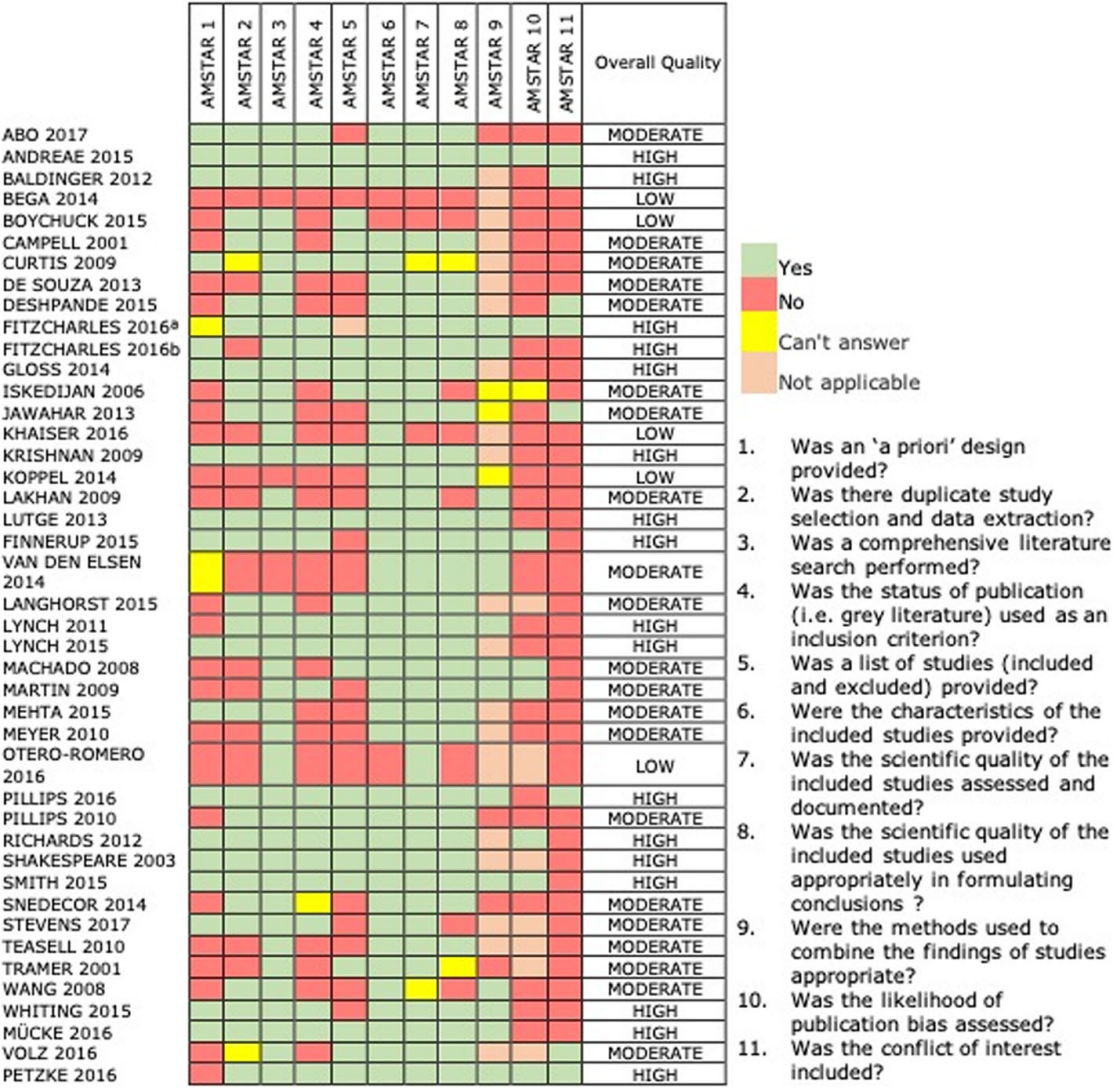
Methodological quality of included Systematic Reviews

Quality of the included SRs according to AMSTAR scores was categorized as “low” in five studies (11,3%) [20–24], as “moderate” in 22 studies [19, 25–44] and as “high” in 17 SRs [1, 45–57] (Fig. 2). The most frequent drawbacks of SRs included no reporting of conflicts of interest, no assessment of publication bias, and absence of ‘a priori design’.

### Characteristics of individual studies

A total of 158 individual studies were analyzed in these SRs, after considering duplication of studies. The number of included studies by review ranged from one [33, 39, 43, 46, 53, 58] to 79 [1]. One-hundred forty-six studies (92,4%) were randomized clinical trials (RCT), of which 59 were parallel and 84 were cross-over trials, and the remaining three studies did not have enough information to define the type of RCT. Two studies (1.2%) were non- randomized clinical trials (NRCT), seven (4,4%) were uncontrolled clinical trials, and three (1.9%) were observational studies.

Most of the individual studies were conducted in the USA (*n* = 57; 36%), followed by the United Kingdom (*n* = 29; 18,3%), and Canada (*n* = 10; 6,4%). Thirteen trials were conducted in more than one country (8,3%). Forty-nine studies were funded by pharmaceuticals companies (31%) while 34 were funded by academic societies (21,9%). Follow-up of participants ranged from 1 day to 48 weeks. One-hundred fifteen studies compared interventions of cannabis or cannabinoids with placebo. Characteristics of individual studies are provided in Additional file 3.

### PICO questions

We extracted 96 PICO questions. PICOs were grouped in the following clinical scenarios. We provided details of PICOs in Additional file 4.

### Multiple sclerosis

The included SRs addressing the management of several symptoms associated to Multiple Sclerosis (MS), including pain, spasticity, bladder dysfunction, and tremor.

The largest number of SRs evaluated the effect of medical cannabis on MS related pain. When cannabinoids in general were compared with placebo, the authors of two SRs (15 RCT) claimed a “probably beneficial” and “unclear” conclusion, respectively [29, 44]. For this indication, there were four different cannabis presentations: oromucosal cannabis spray, oral cannabis’ extract, smoked cannabis and dronabinol, all which were compared with placebo. Two SRs claimed “probably beneficial” [23] and “unclear” [50] conclusions for oromucosal cannabis spray. Oral cannabis’ extract was assessed in one SR and the authors concluded a “beneficial” effect [23]. Two SRs yielded an “unclear” conclusion for smoked cannabis [23, 28]. Finally, in three SRs where the presentation was dronabinol the authors concluded a “probably beneficial” conclusion [23, 29, 30]. Only one SR assessed the efficacy of Nabilone plus Gabapentin versus an active control (Gabapentin alone), the conclusion was “probably beneficial” [50].

When investigating spasticity, cannabinoids were compared with placebo in three SRs (12 RCTs). The authors of two SRs reported a “probably beneficial” conclusion [1, 31], and one an “unclear” conclusion [54]. Additionally, three administration routes were compared with placebo. Oral cannabis extract containing THC / CBD was examined in three SRs (five RCTs). The results of two SRs were reported as “probably beneficial” [23, 50], and in one the conclusion was “unclear” [24]. Oromucosal cannabis spray containing THC / CBD was studied in two SRs (five RCTs and one uncontrolled trial), that reported a “probably beneficial” conclusions [23, 24]. Smoked cannabis was assessed in three SRs (two RCTs), obtaining an “unclear” result [23], and “probably beneficial” conclusions [22, 50]. Finally, one SR compared THC-CBD (including both oral and oromucosal presentations) with THC alone (three RCT), its results were reported as “unclear” [31].

In relation to bladder dysfunction, an SR (two RCTs and one NRCT) compared cannabis and placebo; authors concluded that cannabis is “probably beneficial” [18]. Specific presentations of cannabis were compared with placebo. One SR evaluated oromucosal cannabis spray (two RCTs), which was reported as “probably beneficial” [23]. In another SR, oral cannabis’ extract was assessed in two different formulations: forms containing THC and CBD with data from five RCT, and forms containing THC alone with data from two RCTs. The conclusion for both comparisons was “no effect” [23].

**Fig. 3:**
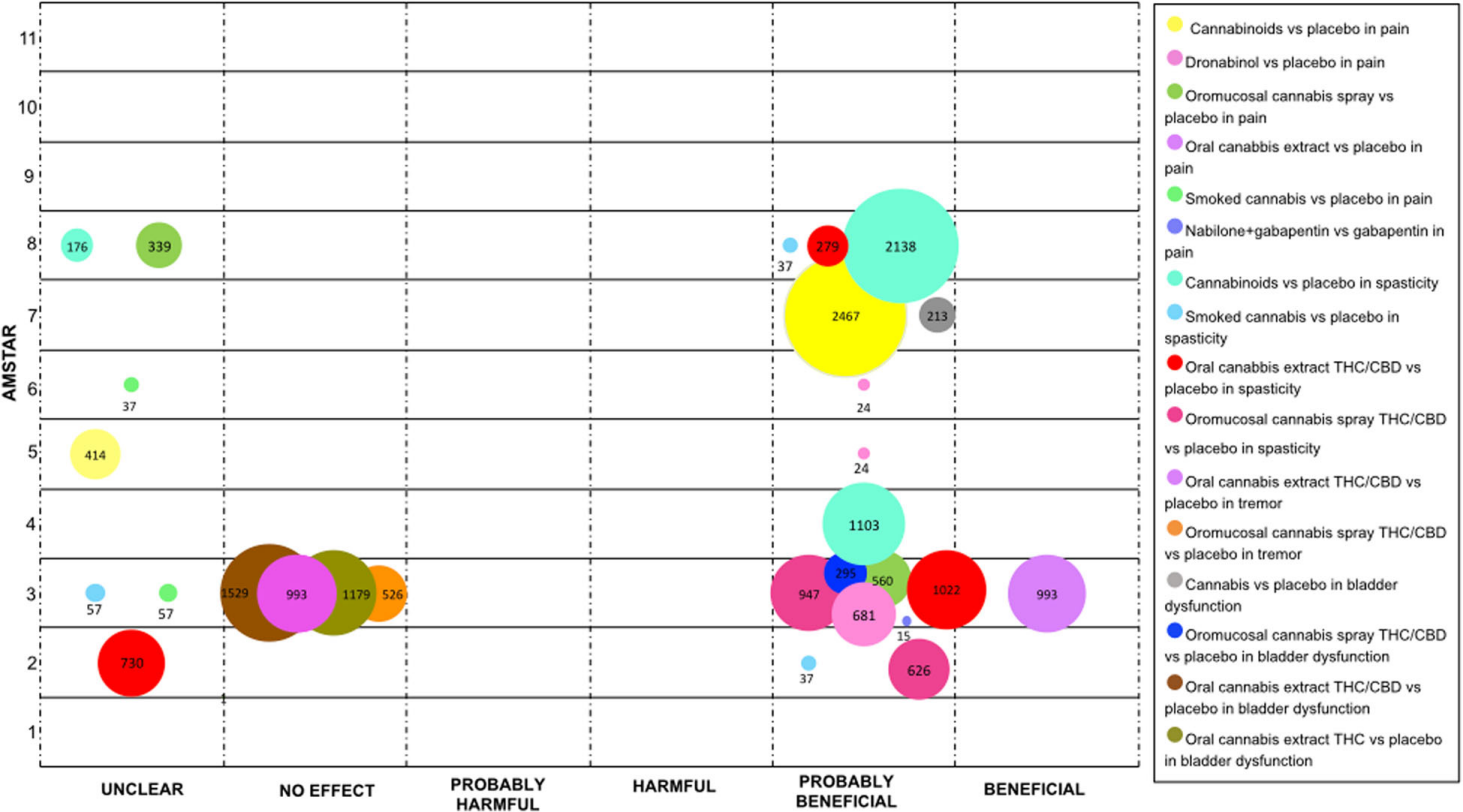
Evidence mapping of cannabis uses in Multiple Sclerosis

Finally, one SR focused on cannabis’ effect on tremor compared two formulations containing THC and CBD with placebo. One formulation was oral cannabis extract (three RCT), and the other was oromucosal cannabis spray (two RCT). For both comparisons, the conclusion was “no effect” [23] (Fig. 3).

### Movement disorders

Cannabinoids have been studied for symptomatic control of various involuntary movement conditions. In terms of Tourette Syndrome, four SRs compared oral cannabinoid (dose 2.5–20 mg) versus placebo (two RCTs), three of them concluded that the effects were “unclear” and one as “probably beneficial” [1, 23, 26, 44].

Regarding Parkinson’s disease, one SR compared cannabis and cannabinoids versus placebo (two RCTs) and reported an “unclear” conclusion [20]. For levodopa-induced dyskinesia, two SRs (one RCT) compared oral THC / CBD versus placebo and stated a “no effect” conclusion [23, 32].

For Huntington’s disease cannabis was evaluated in one SR. This SR compared Nabilone and oral cannabidiol with placebo, each comparison included one RCT. For both comparisons, the authors concluded an “unclear” effect [23].

**Fig. 4:**
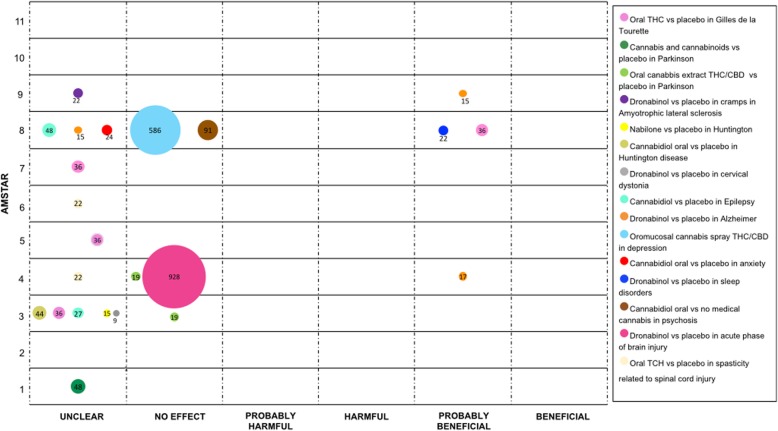
Evidence mapping of cannabis uses in Movement Disorders, psychiatric conditions and other neurological disorders

Finally, the effect of Dronabinol versus placebo for cramps in different conditions was evaluated. One SR (one RCT) evaluated the effect in amyotrophic lateral sclerosis and another SR (one RCT) in Cervical Dystonia. Authors of both SRs reported an “unclear” effect [23, 46]. See Fig. 4.

### Psychiatric conditions

Psychiatric conditions for which cannabis has been studied include: clinical depression, anxiety, sleeping disorders, and psychosis. Evaluating clinical depression, one SR (three RCTs) compared oromucosal cannabis spray containing THC and CBD versus placebo, the authors conclusion was “no effect” [1].

For anxiety disorders, one SR (one RCT), compared oral cannabidiol versus placebo, results were reported as “unclear” [1]. Likewise, for sleeping disorders, one SR (one RCT) contrasted Dronabinol with placebo, showing a “probably beneficial” conclusion [1]. Finally, for psychosis one SR (two RCTs) evaluated oral cannabidiol versus no medical cannabis concluding as “no effect” [1]. See Fig. 4.

### Other neurological disorders

The medical use of cannabis has been assessed in a heterogeneous group of neurologic conditions. Two SRs compared oral Cannabidiol with placebo for patients with epilepsy (three RCTs and one NRCT). Authors of both SRs obtained an “unclear” conclusion [23, 59].

Three SRs (two RCTs) evaluated Dronabinol compared with placebo to treat anorexia, disturbed behavior and agitation in patients with Alzheimer’s disease. The conclusions of the authors were “probably beneficial” in two SRs [32, 56] and “unclear” in one SR. [58]

In patients with an acute phase of acquired brain injury, Dronabinol was compared with placebo to manage intracranial pressure. The comparison was assessed in one SR (two RCTs), and it was concluded that there is “no effect” [37].

For the management of spasticity in patients with spinal cord injury, two SRs (one RCT) compared oral TCH with placebo. Authors concluded the effect to be “unclear” [36, 41]. See Fig. 4.

### Pain in general

There is a large amount of evidence surrounding cannabis and cannabinoids in the management of acute and chronic pain. It has either been studied as an isolated symptom or in association with other diseases (i.e. diabetes mellitus or cancer).

#### Chronic pain

Two SRs (37 RCTs) assessed cannabis and cannabinoids with placebo. One SR reported “probably harmful” effects [35], and one SR reported a “probably beneficial” effects [1]. One SR evaluated cannabinoids versus codeine (two RCTs), results were reported to be “probably harmful” [25]. Vaporized cannabis was also studied for chronic pain relief compared to placebo in one SR. A “probably beneficial” conclusion was found in 21 patients involved in one uncontrolled study [22].

One SR focused on the comparison of cannabinoids’ effects against placebo for chronic pain, not associated with cancer. This review included nine RCTs for cannabis and cannabinoids, four RCTs for smoked cannabis, seven RCTs for oromucosal cannabis spray and two RCTs for dronabinol. The conclusions for these four comparisons were stated as “beneficial” [50]. Furthermore, oral cannabis extract was compared with placebo in one SR, with two patients. The authors stated a “no effect” conclusion [25].

To consider the effects of cannabis and cannabinoids in neuropathic pain, four categories were established: neuropathic pain in general, posttraumatic neuropathic pain, diabetic neuropathy, and neuropathic pain associated with allodynia.

With regards to neuropathic pain in general, three SRs compared cannabis and cannabinoids versus placebo (21 RCTs), conclusions were reported as “beneficial”, “probably beneficial”, and “unclear” [21, 44, 51]. When cannabinoids were compared with placebo three SRs were found (15 RCTs) [29, 49, 57], two SRs concluded that cannabinoids were “probably beneficial” and one as “probably harmful”. When just cannabis was included in comparison, the authors of one SR (four RCTs) stated a “probably beneficial” conclusion [28]. Furthermore, smoked cannabis, vaporized cannabis, oromucosal cannabis spray and CT-3 (an analogue of THC-11-oic acid) were compared with placebo, with “probably beneficial” conclusions for all of these comparisons.

Furthermore, two different cannabis presentations were compared with active compounds in patients with neuropathic pain. One SR assessed oral cannabis extract versus codeine (one crossover RCT with one patient), authors from this SR stated a “probably beneficial” conclusion [25]. Two SRs (one RCT) that compare Nabilone with Dihydrocodeine stated conclusions considered as “no effect” [51, 57].

In relation to doses, two SRs (three RCTs) compared low vs. high dosage of cannabis. Conclusions ranged from “probably beneficial” to “no effect” conclusions [22, 28].

For posttraumatic neuropathic pain, three comparisons were conducted. One SR evaluated smoked cannabis versus placebo (one RCT), the conclusion was “unclear” [28]. Another SR compared Dronabinol with Diphenhydramine (one RCT), the conclusion was “no effect” [36]. Regarding dose, one SR (one RCT) compared low vs. high dosage of cannabis, it was concluded as “unclear” [22].

For diabetic neuropathy, two cannabinoids presentations were compared with placebo. For Nabilone one SR concluded as “probably beneficial” [50]. For cannabis spray (one RCT), the obtained results were considered “unclear” by one SR. [39] In addition, one SR (one RCT) compared high with low doses of vaporized THC, results were considered as “probably beneficial” [22].

Finally, in patients with neuropathic pain associated with allodynia, one SR compared oromucosal cannabis spray with placebo, and concluded as “probably beneficial” [50].

#### Acute pain

Cannabinoids were compared with placebo in one SR (five RCTs). The conclusion from the authors was “unclear” [40]. Likewise, Dronabinol and smoked cannabis were compared with placebo in one SR, with the inclusion of two and one RCT, respectively. The conclusion was rated by the authors as “probably beneficial” for Dronabinol and “unclear” for smoked cannabis [22]. The same SR, based on one RCT, evaluated smoked cannabis versus Dronabinol; it was concluded as “unclear” [22].

The postoperative etiology of acute pain was assessed in two SRs, the intervention of Levonantradol was compared with placebo (two RCTs). One of these SRs showed a “probably beneficial” conclusion [25], while the other presented an “unclear” conclusion [40].

**Fig. 5:**
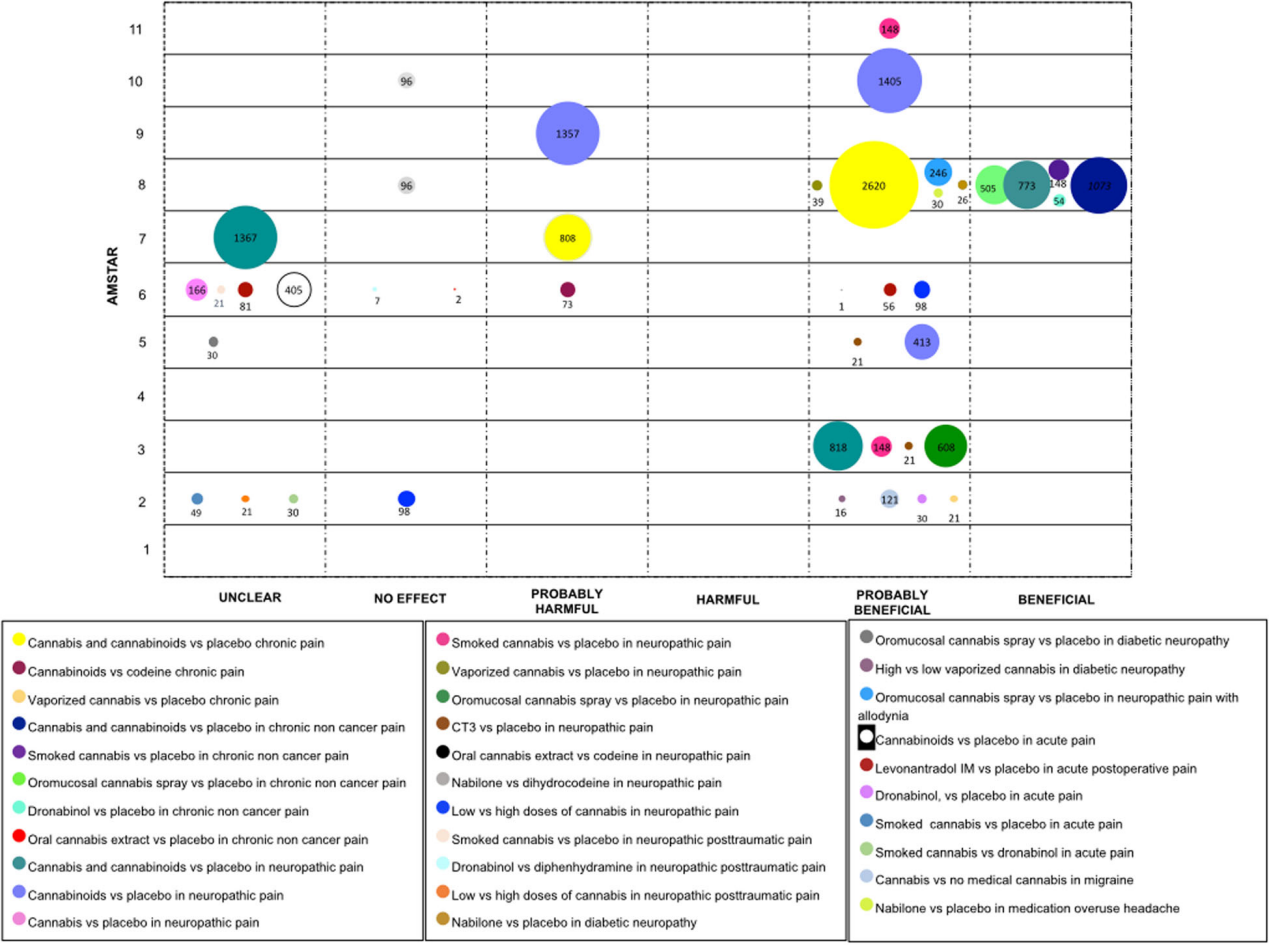
Evidence mapping of cannabis use in pain

Two types of headaches were assessed in two SR. One SR, which included data of one observational study, contrasted cannabis with no medical cannabis for patients with migraine, a “probably beneficial” conclusion was obtained [22]. The other SR compared Nabilone versus placebo in patients with headache due to medication overuse. The conclusion was “probably beneficial” [50]. See Fig. 5.

### Cancer

In patients with cancer, the most frequent symptom studied was emesis induced by chemotherapy, comparisons were performed with placebo and with active controls.

Four SRs compared cannabinoids with placebo (13 RCTs) [1, 42, 44, 55]. All these SRs stated a “probably beneficial” conclusion. One SR conducted a more specific comparison with Dronabinol versus placebo (three RCTs), its conclusion was stated as “unclear” [34].

Cannabinoids were compared with conventional antiemetics in six SRs including a total of 31 RCTs [32, 34, 42, 44, 52, 55]. Two SRs obtained a “probably beneficial” conclusion, two an “unclear” conclusion, and two concluded as “no effect”. Meanwhile, one SR reported a “no effect” conclusion when compared cannabinoids plus antiemetic versus antiemetic alone (two RCTs and two NRCTs) [55].

Two SRs compared Dronabinol versus neuroleptics (four RCTs), one of these SRs concluded as “probably beneficial” and the other as “no effect” [32, 34]. Nabilone and Levonantradol were compared with neuroleptics by one SR (seven RCTs for Nabilone and two RCTs for Levonantradol) that reported an “unclear” conclusion [34].

In relation to anorexia associated with cancer, cannabinoids were compared with placebo in one SR (three RCTs), the conclusion was stated as “no effect” [56]. Likewise, in one SR Dronabinol was compared with Megestrol with data from one RCT, the conclusion was “probably harmful” effect [56].

Regarding cancer pain, one SR compared oral Benzopyranoperidine, oral THC and synthetic nitrogen analogue of THC with placebo. For Benzopyranoperidine, the conclusion from the authors was “no effect” (one RCT). For oral THC, the authors concluded as “probably beneficial” (two RCTs). For synthetic nitrogen analogue of THC (two RCTs), the conclusion was reported as “probably harmful” [25].

This SR also compared the effects of cannabinoids against codeine. In two studies, one RCT for Benzopyranoperidine and one RCT for oral THC, the authors concluded “no effect” in both comparisons. In regards to the synthetic nitrogen analogue of THC based on the data from one RCT, the conclusion was reported as “probably harmful”. Additionally, synthetic nitrogen analogue of THC was compared with secobarbital in one RCT; the conclusion was stated as “probably harmful” [25]. For refractory cancer pain, one SR concluded as “probably beneficial” when compared oromucosal cannabis spray with placebo (two RCT) [56].

**Fig. 6:**
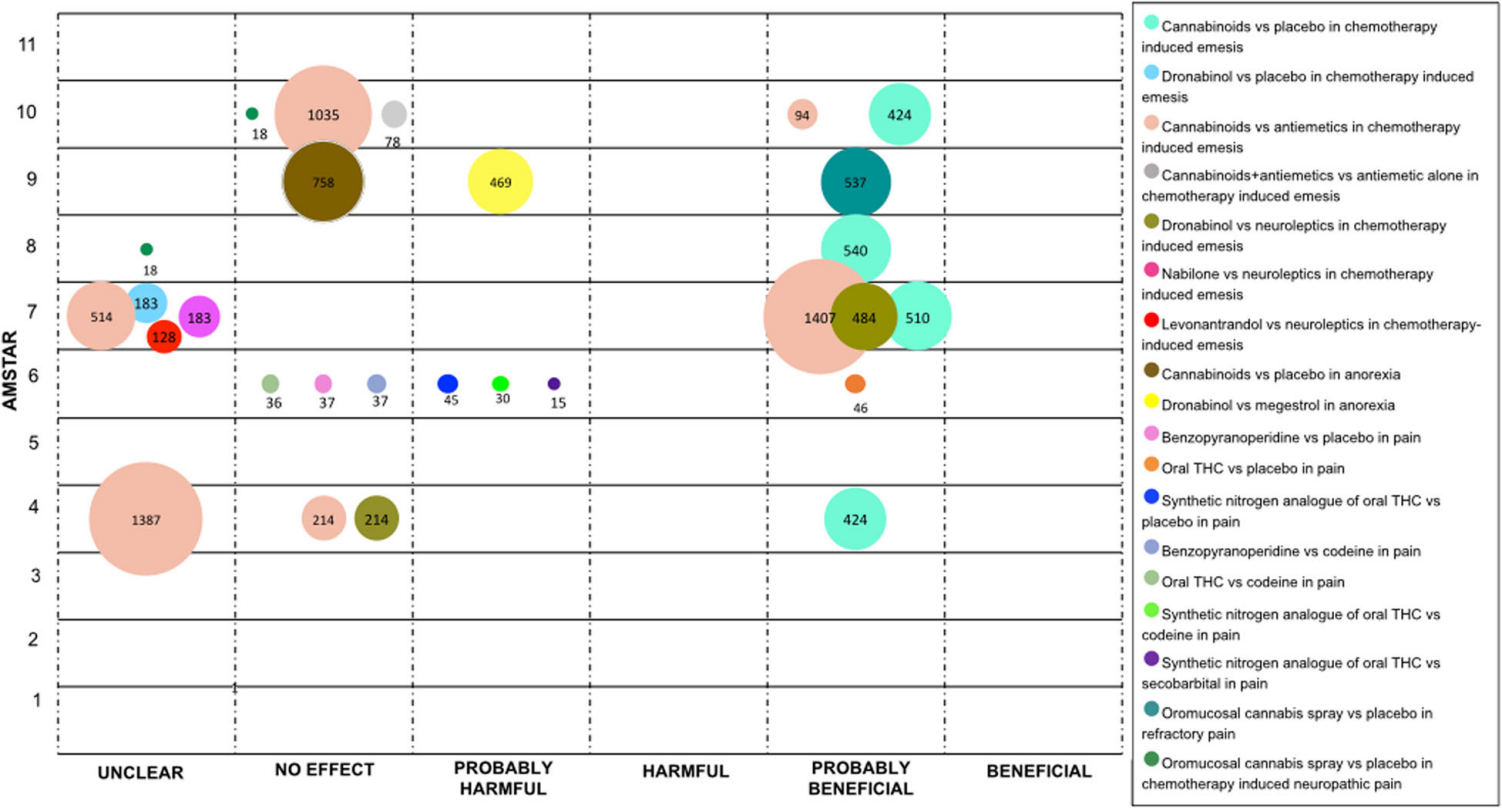
Evidence mapping of cannabis uses in Cancer

Finally, two SRs evaluated oromucosal cannabis spray versus placebo for the management of chemotherapy induced neuropathic pain. These SRs with data from one RCT concluded as “unclear” and “no effect [50, 57]. See Fig. 6.

### Other medical conditions

In this section, we described several conditions which were not included in the previous sections.

The use of medical cannabis has been studied in rheumatic disorders such as rheumatoid arthritis, fibromyalgia, Crohn’s disease, spinal chronic pain, and osteoarthritis. For rheumatoid arthritis, oromucosal cannabis spray was compared against placebo in three SRs (one RCT). Results were reported as “probably harmful”, “unclear”, and “probably beneficial” [47, 48, 53]. For fibromyalgia two comparisons were conducted in four SRs [27, 47, 48, 51] with data from one RCT. When comparing Nabilone versus placebo and Nabilone versus Amitriptyline - three SRs concluded as “probably beneficial” and one as “unclear” for the first comparison and the conclusions were “probably beneficial” in two and “no effect” in the other two SRs for the second comparison. In relation to Crohn’s disease, two SRs compared smoked cannabis with placebo (one RCT), both studies concluded this intervention as “probably beneficial” [33, 43]. For chronic spinal pain, one SR compared Nabilone with placebo (one RCT), its conclusion was reported as “unclear” [48]. Finally, for osteoarthritis of the knee, the PF-04457845, a fatty acid amide hydrolase-1 (FAAH1) inhibitor, was compared with placebo in two SRs (one RCT), in both SRs, authors stated that there was “no effect” [47, 50].

In patients with HIV-AIDS, cannabis and cannabinoids were compared with placebo for general symptoms in three SRs (eight RCTs), conclusions were “unclear” in two, and “probably beneficial” in one [1, 44, 60]. For HIV-related neuropathic pain, smoked cannabis was compared with placebo in three SRs (two RCTs), the conclusions were “unclear” in one and “probably beneficial” in two [22, 38, 51]. Regarding HIV wasting syndrome, three different comparisons were conducted. Herbal cannabis versus synthetic cannabinoids was addressed in one SR (one RCT), the conclusion was “unclear” [56]. Two SRs (five RCTs) compared Dronabinol with placebo and their conclusions were reported as “unclear” and “probably beneficial” [56, 60]. Dronabinol was also compared with Megestrol in one SR (one RCT) and the conclusion was “no effect” [56].

Glaucoma was another condition addressed in one SR (one RCT), where oromucosal cannabis spray was compared with placebo, the conclusion was reported as “unclear” [1].

**Fig. 7:**
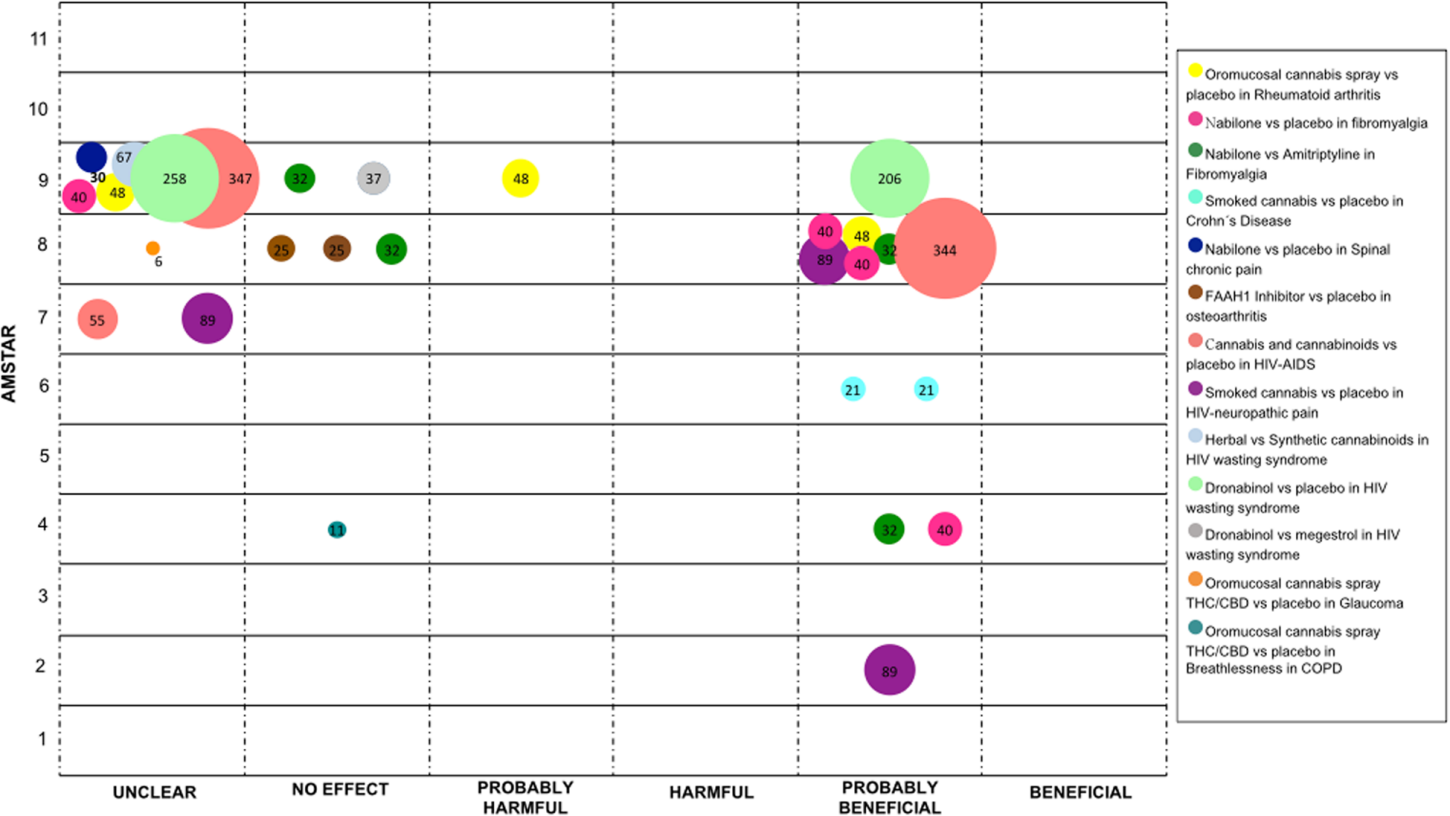
Evidence mapping of cannabis uses in other medical conditions

Finally, in patients with chronic pulmonary obstructive disease, oromucosal cannabis spray was compared with placebo for the management of breathlessness in one SR (one RCT), the conclusion was “no effect” [32]. See Fig. 7.

## Discussion

Our evidence mapping collected information from 44 SRs and 158 studies (most of them RCTs-92.4%) published between 2001 and 2017. The high number of studies reflects the increasing interest by users and physicians in assessing the potential therapeutic value of cannabis for several medical conditions.

We found that effectiveness and safety of medical cannabis has been evaluated in multiple medical conditions such as multiple sclerosis, movement disorders (e.g. Tourette Syndrome, Parkinson Disease), psychiatric conditions, Alzheimer disease, epilepsy, acute and chronic pain, cancer, neuropathic pain, symptoms related to cancer (e.g. emesis and anorexia related with chemotherapy), rheumatic disorders, HIV-related symptoms, glaucoma, and COPD.

Medical conditions addressed by these SRs have been previously identified by surveys about medical cannabis use [59–64]. One of the most representative surveys showed that cannabis was primarily used for back pain (11.9%), sleeping disorders (6.9%), depression (6.7%), pain resulting from injury or accidents (6.2%), and multiple sclerosis (4.1%) [61].

However, we noticed that the evidence for medical cannabis effects on these conditions is heterogeneous regarding the conclusions and the quality of the collected studies. Most of the conclusions extracted from SRs were classified as “probably beneficial” and “unclear”. Furthermore, for some comparisons, conclusions claimed by SRs were inconsistent and even contradictory. One example was the comparison of cannabis and cannabinoids with conventional antiemetics for chemotherapy-induced emesis [32, 34, 42, 44, 52, 55], where two SRs found a “probably beneficial” conclusions, while remaining four SRs claimed for an “unclear” conclusion or “no effect¨.

The evidence supporting the medical use of cannabinoids varies widely by clinical scenarios from high to low quality evidence. In fact, for some medical conditions, that we found in this evidence map, studies cannot reach firm conclusions, although RCTs have been conducted. While for other medical conditions, not showed in this evidence mapping, cannabis has been approved for use with only preliminary data (pre-clinical studies or observational studies) supporting the use, as is the case of hepatitis C, chronic renal failure, and posttraumatic stress disorders [65].

The research on health effects of cannabis and cannabinoids has been limited by regulatory reasons and policies in some countries, leaving patients and health care professionals without the evidence to make decisions regarding the use of cannabis and cannabinoids in local scenarios. Some barriers have been identified to conducting basic, clinical, and population health research on cannabis and cannabinoids, including regulations that restrict access to the cannabis products, funding limitations, and numerous methodological challenges [66].

In relation to funding, we found that most of the analyzed individual studies were sponsored by pharmaceuticals companies. Because of complexity of the research agenda in this field more funding sources and mechanism are needed to better understand the comprehensive health effects of cannabis.

There were also a number of methodologic limitations. The use of reliable placebos and well-selected active control compounds are needed for clinical trials, since the psychoactive and vasoactive effects of cannabis are a considerable challenge for effective blinding [66]. This limitation is important since 71% of the individual studies included in the SRs compared cannabis and cannabinoids against placebo.

Furthermore, restrictions on drug supply lead to the lack of standardization in potency or quantity of pharmacologically active constituents in cannabis products [66, 67]. This barrier leads to another limitation in conducting clinical trials reflecting in the wide variety of cannabis compounds assessed for a given medical condition.

Moreover, to get well-validated evidence it is necessary to have high-quality research. The quality of the SRs was moderate to high according to AMSTAR scores. However, the most frequent drawbacks were: failure to declare conflicts of interest, lack of likelihood of publication bias evaluation and absence of ‘a priori design’. Additionally, it’s important to state that beyond the quality of the SRs, its crucial to judge the quality of the individual primary studies to get a context of what evidence is telling us.

One strength of our evidence mapping is the use of a sensitive and comprehensive search strategy to localize the 44 SRs included as a source of information. We also used a broad definition of SR in order to obtain the largest number of documents. Additionally, this evidence mapping uses a friendly format to organize and classify research questions in PICO format. Findings are shown graphically to allow the identification of research needs, fields of controversy and the overall quality of the SRs included. Interventions were rated according to the conclusions stated by authors of the SRs. It is important to consider that this classification does not represent the effect of the interventions.

One limitation of this evidence mapping is that the quality of the studies included in each SR was not evaluated in addition to the quality of the SRs. Furthermore, as it is a characteristic of evidence mapping methodologies, we did not assess the quality of the evidence supporting the conclusions, which would have required the use of some complementary methodology such as GRADE. In addition, were describe the conclusions of the included studies according to how the authors declared them, however the direction of effects for each comparison should be deeply assessed by systematic reviews. Despite these limitations, this evidence mapping meets its objective of organizing and describing the available evidence as reported by the authors.

## Conclusions

In conclusion, the evidence on medical uses of cannabis is broad and highly heterogeneous. However, due to methodological limitations, conclusions were reported as “probably beneficial” and “unclear” in most of the assessed comparisons. To support the use of cannabis in different clinical conditions additional efforts are needed, as the approval for the use of cannabis and cannabinoids, as any other drug, should rely on well-designed and statistically powered clinical trials.

Evidence mapping methodology is useful to perform an overview of available research, since it is possible to systematically describe the extent and distribution of evidence, and to organize scattered data. This approach helps to identify if there is enough evidence to support policy maker’s decisions, to recognize research-dense areas where systematic reviews can be conducted, and to highlight research priorities in the field. To reach these objectives, SRs are a reliable source of information as they convey comprehensive and appraised data. Furthermore, SRs help to expand or limit the scope of research mapping by modifying the search strategy according to the evidence mapping aims.

## Supplementary information

**Additional file 1:** Search strategies.

**Additional file 2:** Reasons for exclusion.

**Additional file 3:** Characteristics of individual studies included in systematic reviews.

**Additional file 4:** PICOs included on systematic reviews.

## Data Availability

All data generated or analyzed during this study are included in this published article [and its supplementary information files].

## Abbreviations

AMSTAR: Assessing the methodological quality of systematic reviews
CBD: Cannabidiol
COPD: Chronic obstructive pulmonary disease
FAAH1: Fatty acid amide hydrolase-1
HIV: Human immunodeficiency virus
MS: Multiple Sclerosis
NRCT: Non-randomized controlled trial
PICO: Population, intervention, comparison, outcome
RCT: Randomized controlled trial
SRs: Systematic reviews
THC: Tetrahydrocannabinol
UCT: Uncontrolled trial
