# Supplementary material for: Therapeutic use of cannabis and cannabinoids: an evidence mapping and appraisal of systematic reviews
Source: BMC Complement Med Ther. 2020 Jan 15;20:12. doi: 10.1186/s12906-019-2803-2 (PMC7076827; doi:10.1186/s12906-019-2803-2)
Supplement: Supplementary file 1 — Additional file 1: Search strategies. [file 12906_2019_2803_MOESM1_ESM.docx]

**S1 Appendix: Search Strategies**

PubMed

| Search |
| --- |
| #39,"Search (((((((((((((((marijuana[Title/Abstract]) OR marihuana[Title/Abstract]) OR cannab*[Title/Abstract]) OR canab*[Title/Abstract]) OR hashish[Title/Abstract]) OR hash[Title/Abstract]) OR bhang[Title/Abstract]) OR ganja[Title/Abstract]) OR hemp[Title/Abstract]) OR charas[Title/Abstract]) OR ""Cannabis""[Mesh]) OR ""Medical Marijuana""[Mesh])) OR ((((((((((((((((cannador[Title/Abstract]) OR ""Cannabinoids""[Mesh]) OR tetrahydrocannabinol*[Title/Abstract]) OR endocannabino*[Title/Abstract]) OR tetra-hydrocannabinol*) OR nabiximol[Title/Abstract]) OR sativex[Title/Abstract]) OR dronabinol[Title/Abstract]) OR marinol[Title/Abstract]) OR delta-9-tetrahydrocannabinol[Title/Abstract]) OR nabilone[Title/Abstract]) OR dexanabinol[Title/Abstract]) OR cesamet[Title/Abstract]) OR anandamide[Title/Abstract]) OR levonantradol[Title/Abstract]) OR THC[Title/Abstract]))) AND systematic[sb]",716,11:43:23 |
| #40,"Search ",311280,11:39:19 |
| #38,"Search systematic[sb]",311280,11:35:25 |
| #37,"Search (((((((((((((marijuana[Title/Abstract]) OR marihuana[Title/Abstract]) OR cannab*[Title/Abstract]) OR canab*[Title/Abstract]) OR hashish[Title/Abstract]) OR hash[Title/Abstract]) OR bhang[Title/Abstract]) OR ganja[Title/Abstract]) OR hemp[Title/Abstract]) OR charas[Title/Abstract]) OR ""Cannabis""[Mesh]) OR ""Medical Marijuana""[Mesh])) OR ((((((((((((((((cannador[Title/Abstract]) OR ""Cannabinoids""[Mesh]) OR tetrahydrocannabinol*[Title/Abstract]) OR endocannabino*[Title/Abstract]) OR tetra-hydrocannabinol*) OR nabiximol[Title/Abstract]) OR sativex[Title/Abstract]) OR dronabinol[Title/Abstract]) OR marinol[Title/Abstract]) OR delta-9-tetrahydrocannabinol[Title/Abstract]) OR nabilone[Title/Abstract]) OR dexanabinol[Title/Abstract]) OR cesamet[Title/Abstract]) OR anandamide[Title/Abstract]) OR levonantradol[Title/Abstract]) OR THC[Title/Abstract])",42695,11:33:06 |
| #36,"Search (((((((((((((((cannador[Title/Abstract]) OR ""Cannabinoids""[Mesh]) OR tetrahydrocannabinol*[Title/Abstract]) OR endocannabino*[Title/Abstract]) OR tetra-hydrocannabinol*) OR nabiximol[Title/Abstract]) OR sativex[Title/Abstract]) OR dronabinol[Title/Abstract]) OR marinol[Title/Abstract]) OR delta-9-tetrahydrocannabinol[Title/Abstract]) OR nabilone[Title/Abstract]) OR dexanabinol[Title/Abstract]) OR cesamet[Title/Abstract]) OR anandamide[Title/Abstract]) OR levonantradol[Title/Abstract]) OR THC[Title/Abstract]",19790,11:32:56 |
| #35,"Search THC[Title/Abstract]",5492,11:32:15 |
| #34,"Search levonantradol[Title/Abstract]",70,11:30:54 |
| #33,"Search anandamide[Title/Abstract]",3517,11:29:58 |
| #32,"Search cesamet[Title/Abstract]",17,11:28:01 |
| #31,"Search dexanabinol[Title/Abstract]",46,11:27:31 |
| #30,"Search nabilone[Title/Abstract]",240,11:25:59 |
| #29,"Search delta-9-tetrahydrocannabinol[Title/Abstract]",3163,11:25:37 |
| #28,"Search marinol[Title/Abstract]",84,11:24:48 |
| #27,"Search dronabinol[Title/Abstract]",270,11:24:08 |
| #26,"Search sativex[Title/Abstract]",140,11:23:50 |
| #25,"Search nabiximol[Title/Abstract]",2,11:23:32 |
| #24,"Search tetra-hydrocannabinol*",22,11:21:53 |
| #23,"Search endocannabino*[Title/Abstract]",6414,11:20:05 |
| #22,"Search tetrahydrocannabinol*[Title/Abstract]",5784,11:17:52 |
| #20,"Search ""Cannabinoids""[Mesh]",11384,11:15:42 |
| #17,"Search cannador[Title/Abstract]",3,11:13:53 |
| #16,"Search (((((((((((marijuana[Title/Abstract]) OR marihuana[Title/Abstract]) OR cannab*[Title/Abstract]) OR canab*[Title/Abstract]) OR hashish[Title/Abstract]) OR hash[Title/Abstract]) OR bhang[Title/Abstract]) OR ganja[Title/Abstract]) OR hemp[Title/Abstract]) OR charas[Title/Abstract]) OR ""Cannabis""[Mesh]) OR ""Medical Marijuana""[Mesh]",37137,11:11:47 |
| #15,"Search ""Medical Marijuana""[Mesh]",391,11:10:49 |
| #13,"Search ""Cannabis""[Mesh]",7386,11:10:23 |
| #10,"Search charas[Title/Abstract]",25,11:09:26 |
| #9,"Search hemp[Title/Abstract]",628,11:09:16 |
| #8,"Search ganja[Title/Abstract]",40,11:08:01 |
| #7,"Search bhang[Title/Abstract]",28,11:07:51 |
| #6,"Search hash[Title/Abstract]",438,11:07:34 |
| #5,"Search hashish[Title/Abstract]",541,11:07:16 |
| #4,"Search canab*[Title/Abstract]",51,11:06:57 |
| #3,"Search cannab*[Title/Abstract]",25176,11:06:40 |
| #2,"Search marihuana[Title/Abstract]",1055,11:06:08 |
| #1,"Search marijuana[Title/Abstract]",10277,11:05:48 |

The Cochrane Library

ID Search

#1 marijuana:ti,ab,kw

#2 marihuana:ti,ab,kw

#3 cannab*:ti,ab,kw

#4 canab*:ti,ab,kw

#5 hashish:ti,ab,kw

#6 hash:ti,ab,kw

#7 cannador:ti,ab,kw

#8 MeSH descriptor: [Cannabis] explode all trees

#9 MeSH descriptor: [Medical Marijuana] explode all trees

#10 MeSH descriptor: [Cannabinoids] explode all trees

#11 tetrahydrocannabinol*:ti,ab,kw

#12 endocannabino*:ti,ab,kw

#13 tetra-hydrocannabinol*:ti,ab,kw

#14 sativex:ti,ab,kw

#15 dronabinol:ti,ab,kw

#16 marinol:ti,ab,kw

#17 delta-9-tetrahydrocannabinol:ti,ab,kw

#18 nabilone:ti,ab,kw

#19 dexanabinol:ti,ab,kw

#20 cesamet:ti,ab,kw

#21 anandamide:ti,ab,kw

#22 levonantradol:ti,ab,kw

#23 THC:ti,ab,kw

#24 {or #1-#23}
