# Supplementary material for: Therapeutic use of cannabis and cannabinoids: an evidence mapping and appraisal of systematic reviews
Source: BMC Complement Med Ther. 2020 Jan 15;20:12. doi: 10.1186/s12906-019-2803-2 (PMC7076827; doi:10.1186/s12906-019-2803-2)
Supplement: Supplementary file 2 — Additional file 2: Reasons for exclusion. [file 12906_2019_2803_MOESM2_ESM.docx]

**Additional file 2. Reasons for exclusion**

| **Author/year** | **Title** | **REASONS FOR EXCLUSION** |
| --- | --- | --- |
| Ahmed 2016 | Therapeutic use of cannabis in inflammatory bowel disease | Literature search is not described. Evaluation of the quality of the studies not conducted |
| Andrzejewski 2016 | Cannabinoids in the treatment of movement disorders: A systematic review of case series and clinical trials | Evaluation of the quality of the studies not conducted |
| Ashton 2005 | Cannabinoids in bipolar affective disorder: a review and discussion of their therapeutic potential | Evaluation of the quality of the studies not conducted |
| Balog 1998 | HIV wasting syndrome: treatment update | Evaluation of the quality of the studies not conducted. Literature search limited to one database |
| Bao 2014 | Complementary and alternative medicine for cancer pain: an overview of systematic reviews | Overview of systematic reviews |
| Branas 2000 | Treatments for fatigue in multiple sclerosis: a rapid and systematic review | Search strategy conducted to identify reviews |
| Burns 2006 | Cannabinoid analgesia as a potential new therapeutic option in the treatment of chronic pain | Evaluation of the quality of the studies not conducted. Literature search limited to one database |
| CADTH 2012 | Cannabinoids for the treatment of post-traumatic stress disorder: a review of the clinical effectiveness and guidelines (Structured abstract) | The search was described as limited |
| Cahill 2013 | Pharmacological interventions for smoking cessation: an overview and network meta-analysis | Overview of Rimonabat |
| Cahill 2011 | Cannabinoid type 1 receptor antagonists for smoking cessation | Assessed efficacy of rimonabat |
| Carter G2011 | Cannabis in palliative medicine: improving care and reducing opioid-related morbidity | Literature search is not described. Evaluation of the quality of the studies not conducted |
| Castaneto 2014 | Synthetic cannabinoids: epidemiology, pharmacodynamics, and clinical implications | Literature search is not described. Evaluation of the quality of the studies not conducted |
| Cocchetto 1981 | A critical review of the safety and antiemetic efficacy of delta-9-tetrahydrocannabinol | Literature search is not described. Narrative review |
| Cotter 2009 | Efficacy of Crude Marijuana and Synthetic Delta-9-Tetrahydrocannabinol as Treatment for Chemotherapy-Induced Nausea and Vomiting: A Systematic Literature Review | Evaluation of the quality of the studies not conducted |
| Curioni 2006 | Rimonabant for overweight or obesity | Assessed efficacy of rimonabat |
| Davis 2016 | Cannabinoids for symptom management and cancer therapy: The evidence | Literature search is not described. Evaluation of the quality of the studies not conducted |
| Gilron 2014 | Emerging drugs for neuropathic pain | Literature search is not described. Evaluation of the quality of the studies not conducted |
| Grotenhermen 2012 | The therapeutic potential of cannabis and cannabinoids | Evaluation of the quality of the studies not conducted |
| Karila 2016 | The Synthetic Cannabinoids Phenomenon | Literature search is not described. Evaluation of the quality of the studies not conducted |
| Kerbage 2015 | Non-antidepressant long-term treatment in post-traumatic stress disorder (Ptsd) | Literature search is not described. Evaluation of the quality of the studies not conducted |
| Koppel 2015 | Cannabis in the Treatment of Dystonia, Dyskinesias, and Tics | Literature search is not described. Evaluation of the quality of the studies not conducted |
| Kramer 2015 | Medical marijuana for cancer | Evaluation of the quality of the studies not conducted. Literature search limited to one database |
| Leweke 2016 | Therapeutic potential of cannabinoids in psychosis | Evaluation of the quality of the studies not conducted. Literature search limited to one database |
| May 2016 | Dronabinol for chemotherapy-induced nausea and vomiting unresponsive to antiemetics | Literature search is not described. Evaluation of the quality of the studies not conducted |
| Ng 2017 | Symptomatic treatments for amyotrophic lateral sclerosis/motor neuron disease | Systematic review not focused on cannabinoids or cannabis |
| Peng 2016 | Medical marijuana as a therapeutic option for cancer anorexia and cachexia: A scoping review of current evidence | Evaluation of the quality of the studies not conducted |
| Prud'homme 2015 | Cannabidiol as an Intervention for Addictive Behaviors: A Systematic Review of the Evidence | Evaluation of the quality of the studies not conducted. Literature search limited to one database |
| Schroder S2013 | Can medical herbs stimulate regeneration or neuroprotection and treat neuropathic pain in chemotherapy-induced peripheral neuropathy? Evidence-based complementary and alternative medicine : eCAM | Evaluation of the quality of the studies not conducted |
| Tafelski 2016 | Efficacy, tolerability, and safety of cannabinoids for chemotherapy-induced nausea and vomiting--a systematic review of systematic reviews | Systematic review of Systematic reviews |
| Taylor 1998 | Analysis of the medical use of marijuana and its societal implications | Evaluation of the quality of the studies not conducted. Literature search limited to one database. Narrative review |
| Teasell 2010 | A systematic review of pharmacologic treatments of pain after spinal cord injury | Previous version other systematic review included (Metha 2016) |
| Tsang 2016 | Nabilone for the Management of Pain | Evaluation of the quality of the studies not conducted |
| Wade 2010 | Meta-analysis of the efficacy and safety of Sativex (nabiximols), on spasticity in people with multiple sclerosis | Literature search is not described. Evaluation of the quality of the studies not conducted |
| Waldon 2013 | Trials of pharmacological interventions for Tourette Syndrome: A systematic review | Evaluation of the quality of the studies not conducted |
| Wilkinson 2016 | A Systematic Review of the Evidence for Medical Marijuana in Psychiatric Indications | Literature search limited to one database |
| Yang 2016 | Interventions for tic disorders: An overview of systematic reviews and meta analyses | Overview of systematic reviews |
| Hill 2015 | Medical Marijuana for Treatment of Chronic Pain and Other Medical and Psychiatric Problems A Clinical Review | Literature search limited to one database. Narrative review |
| Musty 2001 | Effects of smoked cannabis and oral delta-9-tetrahydrocannabinol on nausea and emesis after cancer chemotherapy: A review of state clinical trials. | Review of the technical reports of the states |
| Gelfand 2006 | Rimonabant: a cannabinoid receptor type 1 blocker for management of multiple cardiometabolic risk factors | Assessed efficacy of rimonabat |
| Fiz 2011 | Cannabis Use in Patients with Fibromyalgia: Effect on Symptoms Relief and Health-Related Quality of Life | Survey |
| Mohamed Ben Amar 2006 | Cannabinoids in medicine: A review of their therapeutic potential | Literature search limited to one database. Narrative Review |
| Burns 2006 | Cannabinoid analgesia as a potential new therapeutic option in the treatment of chronic pain. | Literature search limited to one database. Narrative Review |
| CADTH 2010A | Cannabinoids as Co-Analgesics: Review of Clinical Effectiveness | Evaluation of the quality of the studies not conducted |
| CADTH 2010B | Cannabinoids for the Management of Neuropathic Pain: Review of Clinical Effectiveness | The search was described as limited |
| Rathbone 2008 | Cannabis and schizophrenia | No assessed efficacy |
| Zammit 2008 | Effects of cannabis use on outcomes of psychotic disorders: systematic review | No assessed efficacy |
| Cahill 2007 | Cannabinoid type 1 receptor antagonists (rimonabant) for smoking cessation | Assessed efficacy of rimonabat |
| Gloss 2012 | Cannabinoids for epilepsy | Previous version other systematic review included (Gloss 2014) |
| Phillips 2010 | Antiemetic medication for prevention and treatment of chemotherapy induced nausea and vomiting in childhood | Previous version other systematic review included (Phillips 2016) |
